# Associations between maternal thyroid function in pregnancy and child neurodevelopmental outcomes at 20 months in the Seychelles Child Development Study, Nutrition Cohort 2 (SCDS NC2)

**DOI:** 10.1017/jns.2021.66

**Published:** 2021-08-31

**Authors:** Anna M. Monaghan, Maria S. Mulhern, Emeir M. Mc Sorley, J.J. Strain, Theresa Winter, Edwin van Wijngaarden, Gary J. Myers, Philip W. Davidson, Conrad Shamlaye, Jude Gedeon, Alison J. Yeates

**Affiliations:** 1 Nutrition Innovation Centre for Food and Health, Ulster University, Coleraine, UK; 2 Institute of Clinical Chemistry and Laboratory Medicine, University Medicine Greifswald, Germany; 3 Department of Community and Preventive Medicine, University of Rochester School of Medicine and Dentistry, Rochester, NY, USA; 4 Department of Pediatrics, University of Rochester School of Medicine and Dentistry, Rochester, NY, USA; 5 Department of Neurology, University of Rochester School of Medicine and Dentistry, Rochester, NY, USA; 6 Minsitry of Health, Mahe, Republic of Seychelles; 7 Child Development Centre, Ministry of Health, Mahe, Republic of Seychelles

**Keywords:** Cognitive development, Iodine, Thyroid function, FFQ, Food Frequency Questionnaire, fT3, free triiodothyronine, fT4, free thyroxine, MDI, Mental Developmental Index, PDI, Psychomotor Developmental Index, TSH, thyroid-stimulating hormone

## Abstract

Maternal thyroid hormones facilitate optimal foetal neurodevelopment; however, the exact role of the thyroid hormones on specific cognitive outcomes is unknown. The present study aimed to investigate associations between maternal thyroid function and neurodevelopmental outcomes in the Seychelles Child Development Study (SCDS) Nutrition 2 cohort (*n* 1328). Maternal free thyroid hormones (fT3, fT4 and fTSH) were assessed at 28 weeks’ gestation with a range of child cognitive outcomes analysed at 20 months. Dietary iodine intake was analysed for a subset of women through a Food Frequency Questionnaire. Linear regression analysis was used to test associations between serum concentrations of maternal thyroid hormones and child neurodevelopment outcomes. Thyroid hormones were analysed as continuous data and categorised as quintiles. 95% of mothers had optimal thyroid function based on fTSH concentrations. Overall, the present study shows that maternal thyroid function is not associated with neurodevelopmental outcomes in this high fish-eating population. However, a positive association, using quintiles for fT3, was reported for the Mental Developmental Index, between Q3 *v.* Q4 (*β* 0⋅073; *P* 0⋅043) and for Q3 *v*. Q5 (*β* value 0⋅086; *P* 0⋅018). To conclude, mothers in our cohort, who largely have optimal thyroid function and iodine intakes, appear able to regulate thyroid function throughout pregnancy to meet neurodevelopmental needs. However, it is possible that minor imbalances of fT3, as indicated from our secondary analysis, may impact offspring neurodevelopment. Further investigation of the relationship between maternal thyroid function and infant neurodevelopment is warranted, particularly in populations with different dietary patterns and thereby iodine intakes.

## Introduction

Iodine is an essential micronutrient required for the production of the thyroid hormones, triiodothyronine (T3) and thyroxine (T4), which are necessary throughout pregnancy for adequate foetal neurodevelopment^(1)^. During pregnancy, the maternal thyroid is stimulated by elevated thyroxine-binding globulin to produce more thyroid hormones as a result of foetal and placental demand^(2,3)^. Demand is further exacerbated by the immaturity of the foetal thyroid gland and although this changes during mid-gestation, dependency upon maternal iodine stores remains owing to the rapid transfer of iodide across the placenta in tandem with the high foetal thyroidal turnover^(1,4–6)^. As pregnancy progresses, mothers experience enhanced renal iodine clearance and glomerular filtration rate, making the maintenance of adequate foetal and maternal iodine status more complex^(3)^. The World Health Organization (WHO) recommends an increased requirement for iodine of 250 μg/d during pregnancy, which differs from the 150 μg/d recommended for the general population^(5)^. Clinically, iodine deficiency during pregnancy is defined as urinary iodine concentrations (UIC) of <150 μg/l^(5)^.

Thyroid hormones play a crucial role in foetal neurodevelopment. During the second half of the first trimester, an adequate maternal supply of T4 is required to initiate neuronal proliferation and migration in the cerebral cortex^(7)^. Throughout the second trimester, there is a surge in maternal thyroid hormones alongside a decline in thyroid-stimulating hormone (TSH) that facilitates both growth and neurodevelopmental mechanisms in the developing foetus^(8)^. Post-natal neurodevelopment including further neuronal proliferation is reliant upon adequate amounts of maternal thyroid hormones transferred throughout each trimester^(9)^.

During early pregnancy, maternal fT4 is the sole source of thyroid hormones, with various studies reporting early pregnancy as a critical window for foetal neurodevelopment with lower intelligence scores determined in later testing of the offspring^(1,10–14)^. Studies focused on this period of early gestation (<20 weeks) have also reported that suboptimal early pregnancy thyroid hormone concentrations may be associated with expressive language delay, congenital malformations and increased likelihood of problematic behaviour throughout childhood^(13)^.

Extensive research into the role of iodine in both growth and neurodevelopment has confirmed that severe deficiency throughout pregnancy is clinically manifested as cretinism amongst children (e.g. stunted growth and abnormal cognitive functioning)^(15)^. However, there is also evidence from several countries to suggest negative implications of mild maternal iodine deficiency during pregnancy. Indeed, the Avon Longitudinal Study of Parents and Children (ALSPAC) in the UK found that suboptimal iodine intake resulted in poor cognitive outcomes relating to verbal IQ, reading accuracy and reading comprehension; these poor outcomes were observed amongst the assessed cohort having a median UIC of 91⋅1 μg/l (IQR 53⋅8–143 μg/l)^(16)^. Furthermore, the Norwegian Mother and Child Cohort Study (MoBa), correlated decreased iodine intakes throughout pregnancy (defined as the mildly deficient range of ~120 μg/d) to child language delay, increased behaviour problems and reduced fine motor skills^(17)^.

The effect of excess iodine on thyroid function is classified as the Wolff Chaikoff effect, a protective mechanism in place to protect against the overproduction of thyroid hormones, as excess iodine results in an inhibitory action on T4 synthesis^(18)^. Adults are typically able to overcome the effect and thereby protect against the development of hypothyroidism, but the immature neonatal gland cannot^(18)^. As such the foetus is more susceptible to hypothyroidism and thereby longer-term consequences on thyroid function^(18)^.

A paper published, in 2018, found that dietary iodine intakes at both ends of the spectrum throughout pregnancy were associated with poorer childhood neurodevelopment outcomes at the 18-month stage; intakes of <220 μg/d as well as >391 μg/d resulted in lower cognitive, language and motor scores^(19)^. Indeed, several studies support this work and the importance of optimal thyroid hormone status throughout pregnancy particularly that of T4, in relation to cognitive development, and have concluded that there is an optimum range of concentrations required^(20–22)^. These findings should be viewed with caution but suggest that a delicate balance regarding thyroid function and hormone concentrations is required throughout gestation^(22)^. The intake of iodine during pregnancy, however, required to maintain TSH and T4 concentrations for optimal neurodevelopment remains unknown.

The Seychellois population consume fish frequently in their diet averaging up to nine portions a week and fish is one of the richest sources of iodine in the diet^(23,24)^. Additionally, as part of global efforts to eliminate iodine deficiency disorders, the Seychellois Government has adopted the Universal Salt Iodisation (USI) programme, whereby iodised salt is added both at the manufacturing stage and for home use in ppm (parts per million)^(25)^. Subsequently, it has been assumed that the Seychellois population possess sufficient and potentially even excess iodine intakes^(26)^.

The aim of the present study is to investigate associations between maternal thyroid function and neurodevelopmental outcomes at 20 months of age in a high fish consuming population.

We measured thyroid hormones in Seychellois women at 28 weeks’ gestation during their third trimester and administered a test battery to the children at 20 months of age.

## Subjects and methods

### Study population

**Fig. 1.**
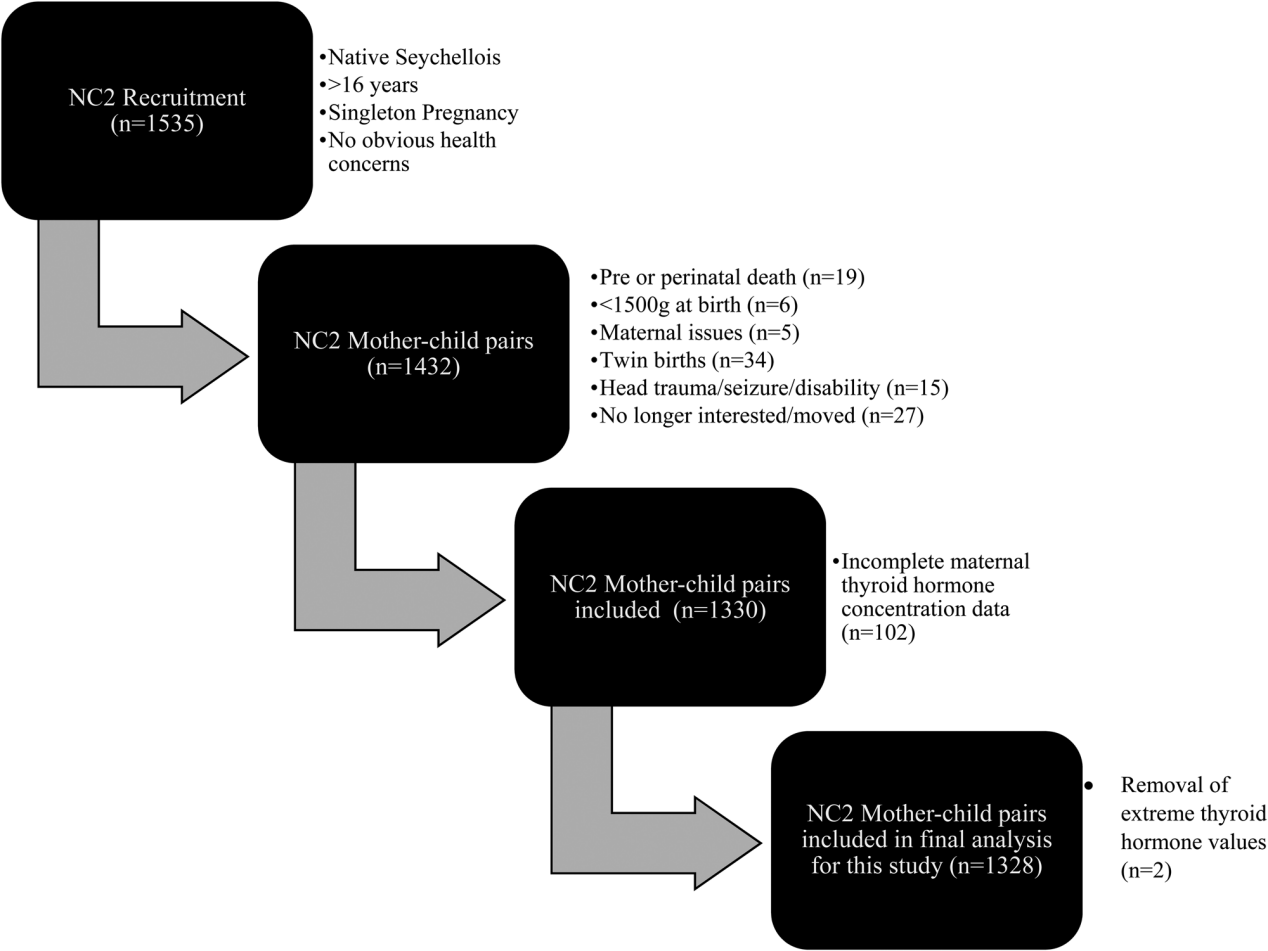
Recruitment process for mother–child pairs within the SCDS NC2.

The Seychelles Child Development Study is centred in the Seychelles, a group of islands off the coast of Africa and has been described previously elsewhere^(27)^. The present study was conducted according to the guidelines laid down in the Declaration of Helsinki. Ethical approval for all procedures involving human participants was obtained from the Seychelles Ethics Board and the Research Subject Review Board at the University of Rochester. Written informed consent was obtained from all participants. The recruitment process of the NC2 cohort is detailed in Fig. 1^(27)^.

### Analytical procedures

For the evaluation of thyroid function, concentrations of the ‘free’ thyroid hormones, denoted as fTSH, fT3 and fT4, were measured. Non-fasting maternal blood samples (30 ml) were taken at 28 weeks’ gestation. The samples were processed immediately (collection by antecubital venepuncture into evacuated serum tubes, placed onto water ice and allowed to sit for 30 min prior to being centrifuged at 2500 rpm for 15 min) with the aliquots stored at −80°C until analysis. Serum aliquots were shipped to Ulster University before undergoing analysis at the Institute of Clinical Chemistry and Laboratory Medicine (IKCL) at the University Medicine Greifswald, Germany. From serum samples, TSH, free triiodothyronine (fT3) and free thyroxine (fT4) were analysed on the Dimension Vista® 1500 System (Siemens Healthcare Diagnostics, Eschborn, Germany). All analyses were performed as single measurements. TSH, fT3 and fT4 were analysed using Siemens chemiluminescent immunoassay assays based on LOCI® technology (REF K6412, K6416, K6410). Concentrations of fT4 are considered the best determinant of thyroid function, but pregnancy-specific reference ranges have yet to be established. Presently, maternal TSH within the reference range of 0⋅3–3⋅0 mIU/l is referred to as optimal TSH based on recommendations by the American Thyroid Association (ATA)^(28)^. Values outside of this range are referred to as suboptimal.

### Test battery

Tests for the children included the Bayley Scales of Infant Development (BSID-II) that yields a Mental Developmental Index (MDI) and a Psychomotor Developmental Index (PDI); The McArthur-Bates Communicative Development Inventory (CDI) that yields scores for total gestures, vocabulary understood and vocabulary produced; and the Infant Behaviour Questionnaire (IBQ-R) that gives scores for negative affect, effortful control and surgency (a personality factor characterised by quickness and cleverness). These three tests assess a range of verbal and non-verbal developments of the offspring including language, communication and social interaction^(29–33)^.

The MDI assesses cognition through the evaluation of sensory perception, knowledge, memory, problem solving and early language. The PDI assesses the psychomotor development of offspring and is viewed in tandem with the MDI as a means of detailing children's overall cognitive development^(29)^. The MacArthur-Bates Communicative Development Inventory (CDI) measures expressive and receptive language functions^(30,31)^. The IBQ measures behavioural development^(34)^. Maternal and Child Health nurses with special training in assessing child development administered the test battery to offspring and their parents at approximately 20 months of age.

### Iodine intake

Nutritionists at Ulster University designed a Food Frequency Questionnaire (FFQ) specifically for use in the Seychelles. At 28 weeks’ gestation, specially trained registered nurses administered this FFQ to a subset of pregnant women (*n*  422) to assess dietary intakes^(33)^. The questionnaire asked participants to specify their average consumption of foods, including various fish species, over the previous 6 months. It included a total of 137 questions on a range of food items. Completed data from the FFQs were entered into QBuilder software (Tinuviel, version 4.0, Anglesey, UK) to estimate iodine intakes (μg/d) and was adapted to incorporate specific Seychellois food items using the Seychellois food composition database.

### Statistics

Maternal and child characteristics were examined, and all data were treated as continuous except for child sex. Based on the Kolmogorov–Smirnov and Shapiro–Wilk tests, the data did not have a normal distribution and so were log-transformed prior to analysis. The removal of two participants with extreme values for both fT3 and fT4 resulted in *n* 1328 mothers for inclusion.

Multiple linear regression models were used to examine the associations between maternal fT4, fT3 and fTSH concentrations and the children's neurodevelopmental tests, first as continuous data. In addition, secondary linear regression models were used to assess non-linear relationships, as evidence suggests that extremes of thyroid hormones may impact cognitive scores^(20)^. Concentrations of fT3, fT4 and fTSH were categorised into quintiles to test their relationships with cognitive data. Quintile 3 was used as the normal reference value for all thyroid hormones (Table 1).

**Table 1.** Quintile concentrations of maternal thyroid hormones (fT4, fT3 and fTSH) measured in the SCDS NC2 (*n* 1328)

Quintile	fT4 (μmol/l)	fTSH (mIU/l)	fT3 (μmol/l)
Q1	<10⋅30 (*n* 246)	<0⋅82 (*n* 264)	<3⋅32 (*n* 262)
Q2	10⋅30–11⋅19(*n* 268)	0⋅82–1⋅09 (*n* 277)	3⋅32–3⋅58 (*n* 269)
Q3 (Ref)	11⋅20–12⋅09 (*n* 277)	1⋅09–1⋅39 (*n* 262)	3⋅59–3⋅84 (*n* 259)
Q4	12⋅10–13⋅00 (*n* 277)	1⋅40–1⋅80 (*n* 266)	3⋅85–4⋅40 (*n* 269)
Q5	>13⋅00 (*n* 260)	>1⋅80 (*n* 259)	>4⋅41(*n* 269)

fTSH, free thyroid-stimulating hormone; mIU/l, milliunits per litre; fT4, free thyroxine; μmol/l, micromoles per litre; fT3, free triiodothyronine.

Given the proportion of our cohort who completed the FFQ (*n* 422; 32 %) and the limitations of dietary data, we chose to use the iodine intake data estimated from the FFQ for descriptive purposes only (Table 2).

**Table 2.** Study characteristics of mothers in the SCDS

	*N*	Median	25th percentile	75th percentile	Max.	Min.
Maternal age (years)	1534	25⋅91	21⋅90	31⋅19	46⋅56	14⋅82
Maternal BMI (kg/m^2^)	1408	26⋅14	21⋅91	30⋅97	49⋅65	14⋅82
Gestational Age (weeks)	1473	39⋅00	38⋅00	40⋅00	41⋅00	26⋅00
Pre-term births	111	–	–	–	–	–
Mother's IQ (KBIT)	1475	30⋅00	25⋅00	35⋅00	−9⋅00	46⋅00
Iodine intake (μg/d)	422	233⋅00	153⋅75	387⋅50	1503	33
Family status	1473	1⋅00	0⋅00	1⋅00	0⋅00	1⋅00
Hollingshead SES	1473	31⋅50	24⋅00	39⋅50	63⋅00	11⋅00
Child age at testing	1472	20⋅00	20⋅00	21⋅00	32⋅00	15⋅00
fTSH (mIU/l)	1329	1⋅24	0⋅90	1⋅68	16⋅80	0⋅25
fT4 (μmol/l)	1330	11⋅60	10⋅50	12⋅70	16⋅80	0⋅03
fT3 (μmol/l)	1330	3⋅73	3⋅38	4⋅06	32⋅80	1⋅24

BMI, body mass index; IQ (KBIT), Intelligence Quotient (Kaufman Brief Intelligence Test); SES, socio-economic status; fTSH, free thyroid-stimulating hormone; μg/d, micrograms per day; mIU/l, milliunits per litre; fT4, free thyroxine; μmol/l, micromoles per litre; fT3, free triiodothyronine.

### Covariates

All models were adjusted for covariates measured within the present study which are known to be associated with child neurodevelopmental outcomes. These were selected *a priori* as in previous analyses of this cohort. Covariates included maternal age at enrolment; maternal BMI at 20 months; gestational age; child sex, child age at testing and, as reported by mothers during pregnancy using questionnaires, maternal IQ using K-BIT; and family status and Hollingshead SES^(24)^.

Statistical analysis was conducted using Statistical Package for Social Sciences (SPSS, version 25, IBM, Chicago, IL, USA). Taking into consideration the covariates outlined, the total number of women included in the adjusted analyses was *n* 1328. The level for determining statistical significance for the two-tailed tests was set at *P* < 0⋅05.

## Results

Table 2 presents a summary of data for participant characteristics. The median age of mothers included was 25⋅91 years with the median gestational age of 39 weeks; a total of 111 premature births (<36 weeks) were recorded. Neurodevelopmental testing was conducted on children between 15 and 32 months (median of 20 months). Women included in the study had a median BMI of 26⋅14 kg/m^2^, classifying them in the slightly overweight category. For the subset of mothers who completed the FFQ (*n* 422), their median iodine intake was 233 μg/d, slightly below the recommended iodine intake for pregnancy as advised by the WHO (>250 μg/d)^(5)^. Based on available TSH measurements within our cohort (*n* 1328), 95 % (*n* 1259) had concentrations within the range of 0⋅3 and 3⋅0 mIU/l, referred to as optimal thyroid function^(28)^. A total of 70 women (5 %) had TSH concentrations either above or below this range, classifying them as having suboptimal (<0⋅3 mIU/l) or supraoptimal (>3⋅0 mIU/l) thyroid hormone status. The T4 concentrations of these seventy women were also analysed with respect to the current reference range during pregnancy of 9⋅0–25⋅0 μmol/l^(28)^. Only one individual displayed suboptimal T4 and TSH concentrations, potentially indicative of a more serious thyroidal condition. For this analysis, all women were included irrespective of whether their TSH was classified as suboptimal or supraoptimal.

As shown in Table 3, there were no significant associations overall between maternal concentrations of fT3, fT4 or fTSH and child neurodevelopmental outcomes, when adjusting for the selected covariates as outlined.

**Table 3.** Associations between maternal concentrations of fT3, fT4 and fTSH with child neurodevelopment outcomes^a^

	fT3 (μmol/l)			fT4 (μmol/l)			fTSH (mIU/l)		
ND test	*β*	95 % Cl	*P* value	*β*	95 % Cl	*P* value	*β*	95 % Cl	*P* value
PDI	−0⋅11	(−10⋅726, 7⋅241)	0⋅704	−0⋅049	(−18⋅276, 1⋅560)	0⋅099	−0⋅005	(−2⋅787, 2⋅326)	0⋅860
MDI	−0⋅031	(−13⋅946, 4⋅024)	0⋅279	−0⋅032	(−15⋅514, 4⋅430)	0⋅276	0⋅001	(−2⋅519, 2⋅620)	0⋅969
IBQ Surg	−0⋅004	(−0⋅662, 0⋅585)	0⋅904	−0⋅041	(−1⋅208, 0⋅197)	0⋅158	0⋅006	(−0⋅161, 0⋅200)	0⋅831
IBG Neg Affect	0⋅003	(−0⋅788, 0⋅875)	0⋅918	0⋅005	(−0⋅857, 1⋅018)	0⋅866	−0⋅031	(−0⋅370, 0⋅112)	0⋅293
IBQ Effortful Control	−0⋅026	(−0⋅878, 0⋅336)	0⋅381	−0⋅04	(−1⋅162, 0⋅206)	0⋅171	0⋅026	(−0⋅094, 0⋅258)	0⋅361
CDI Part l Vocab Prod	0⋅008	(−61⋅376, 81⋅406)	0⋅783	−0⋅013	(−100⋅973, 64⋅398)	0⋅664	−0⋅001	(−21⋅535, 20⋅924)	0⋅978
CDI Part l Vocab Understand	0	(−74⋅428, 75⋅178)	0⋅992	0⋅003	(−81⋅382, 89⋅442)	0⋅926	−0⋅05	(−23⋅975, 19⋅881)	0⋅855
CDI Part l Total Gestures	−0⋅013	(−8⋅847, 5⋅299)	0⋅650	−0⋅02	(−10⋅487, 5⋅127)	0⋅501	0⋅015	(−1⋅469, 2⋅540)	0⋅600

ND, neurodevelopment test; mIU/l, milliunits per litre; μmol/l, micromoles per litre; fT4, free thyroxine; fT3, free triiodothyronine; fTSH, free thyroid-stimulating hormone; MDI, Mental Development Index; PDI, Psychomotor Development Index; IBQ Surg, Infant Behaviour Record-Revised Surgency; IBQ Neg Aff, Infant Behaviour Record-Revised Negative Affect; IBQ Effor Cont, Infant Behaviour Record-Revised Effortful Control; CDI Part l Voca Prod, MacArthur-Bates Communicative Development Inventories Vocab Produced; CDI Part l Voca Und, MacArthur-Bates Communicative Development Inventories Vocab Understand; CDI Part l Total Ges, MacArthur-Bates Communicative Development Inventories Total Gestures.

a Data were log transformed.

**Fig. 2.**
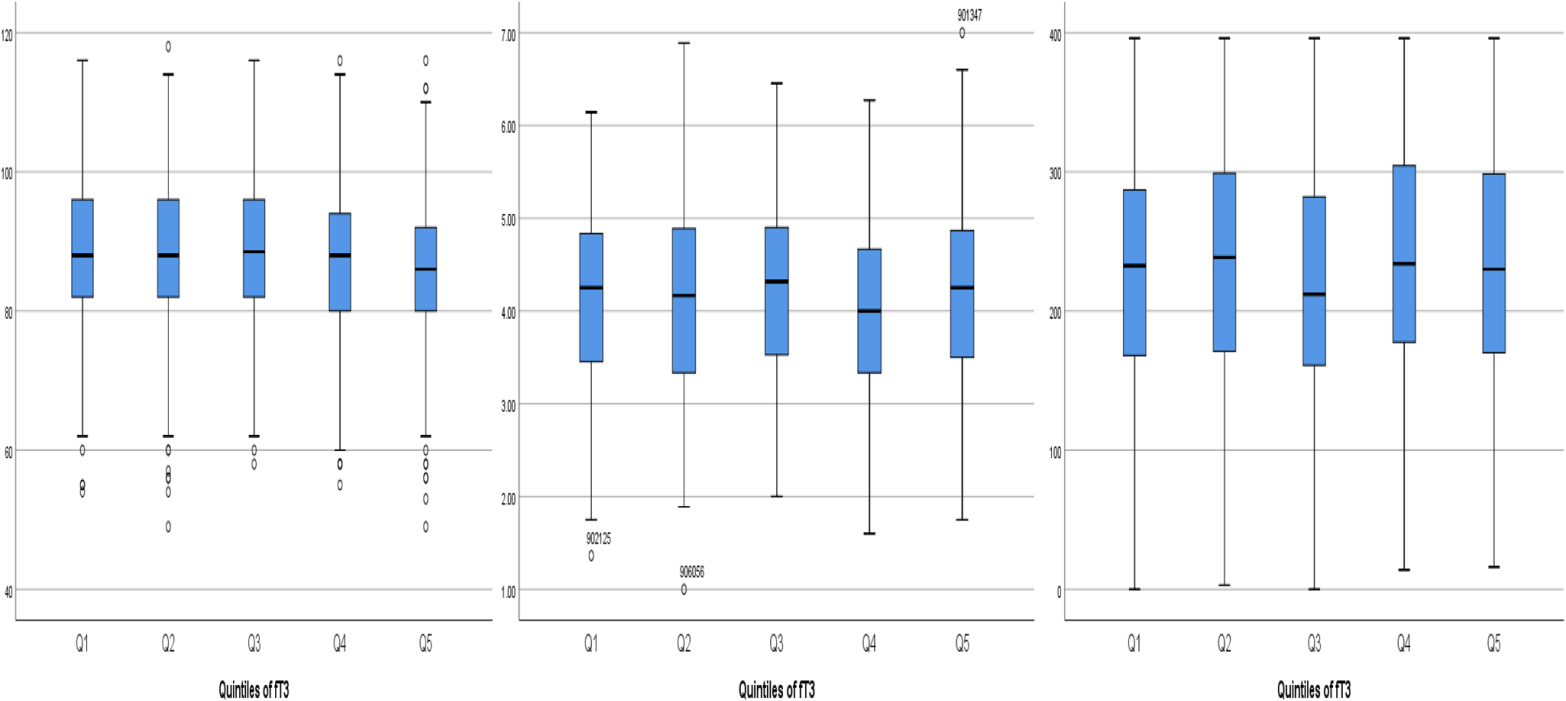
Boxplots of the positive associations between the three cognitive tests MDI, IBQ Negative Affect and CDI Part l Understand and the quintiles of fT3. MDI, Mental Development Index; fT3, triiodothyronine; IBQ Negative Affect, Infant Behaviour Questionnaire Negative Affect; CDI Part l Vocab Understand, Communicative Development Inventory Vocabulary Understand.

Results of analyses examining relationships with quintiles of each thyroid hormone (fT4, fT3 and fTSH) are shown in Tables 4–6. Table 4 depicts fT4, with no associations found in this model. As shown in Table 5, our results indicate that for both Q4 and Q5 of fT3, there is a positive significant association with MDI, compared to the middle quintile (reference) Q3, for Q4 (*β* 0⋅073; *P* 0⋅043) and for Q5 (*β* 0⋅086; *P* 0⋅018). This difference suggests that higher concentrations of fT3 when compared to the reference quintile and thereby lower concentrations were associated with improved scores on the MDI. Table 6 illustrates fTSH, which like fT4 showed no association in this model. These results are illustrated by box plots in Fig. 2.

**Table 4.** Associations between maternal fT4 quintiles and child neurodevelopment outcomes at 20 months of age in the cohort of 1328^a^

	Q1 (*n* 246)			Q2 (*n* 268)			Q4 (*n* 277)			Q5 (*n* 260)		
ND test	*β*	95 % Cl	*P* value	*β*	95 % Cl	*P* value	*β*	95 % Cl	*P* value	*β*	95 % Cl	*P* value
PDI	−0⋅012	(−2⋅245, 1⋅570)	0⋅729	−0⋅026	(−2⋅545, 1⋅178)	0⋅471	0⋅015	(−1⋅449, 2⋅206)	0⋅684	0⋅011	(−1⋅585, 2⋅181)	0⋅756
MDI	−0⋅011	(−2⋅213, 1⋅616)	0⋅760	0⋅023	(−1⋅249, 2⋅479)	0⋅518	0⋅003	(−1⋅756, 1⋅908)	0⋅935	0⋅040	(−0⋅806, 2⋅976)	0⋅260
IBQ Surg	−0⋅018	(−0⋅170, 0⋅101)	0⋅620	−0⋅018	(−0⋅166, 0⋅098)	0⋅616	0⋅050	(−0⋅038, 0⋅222)	0⋅164	0⋅008	(−0⋅119, 0⋅148)	0⋅827
IBG Neg Affect	−0⋅013	(−0⋅214, 0⋅147)	0⋅717	−0⋅03	(−0⋅250, 0⋅102)	0⋅408	0⋅002	(−0⋅168, 0⋅178)	0⋅954	−0⋅038	(−0⋅273, 0⋅083)	0⋅296
IBQ Effortful Control	−0⋅019	(−0⋅169, 0⋅095)	0⋅585	0⋅012	(−0⋅106, 0⋅151)	0⋅735	0⋅023	(−0⋅086, 0⋅167)	0⋅531	0⋅045	(−0⋅046, 0⋅214)	0⋅206
CDI Part l Vocab Prod	−0⋅014	(−19⋅096, 12⋅856)	0⋅702	0⋅005	(−14⋅462, 16⋅644)	0⋅891	0⋅022	(−10⋅477, 20⋅162)	0⋅534	0⋅010	(−13⋅477, 17⋅963)	0⋅780
CDI Part l Vocab Understand	−0⋅001	(−16⋅603, 16⋅363)	0⋅989	0⋅01	(−13⋅736, 18⋅357)	0⋅778	0⋅057	(−2⋅986, 28⋅595)	0⋅112	0⋅002	(−15⋅706, 16⋅732)	0⋅951
CDI Part l Total Gestures	−0⋅018	(−1⋅891, 1⋅122)	0⋅617	0⋅013	(−1⋅201, 1⋅733)	0⋅722	0⋅054	(−0⋅322, 2⋅565)	0⋅128	0⋅005	(−1⋅370, 1⋅595)	0⋅882

ND, neurodevelopment test; fT4, free thyroxine; Q1: <10⋅3 μmol/l; Q2: 10⋅3–11⋅19 μmol/l; Q3: 11⋅20–12⋅09 μmol/l; Q4: 12⋅10–13⋅00 μmol/l; Q4: >13⋅00 μmol/l; MDI, Mental Development Index; PDI, Psychomotor Development Index; IBQ Surg, Infant Behaviour Record-Revised Surgency; IBQ Neg Aff, Infant Behaviour Record-Revised Negative Affect; IBQ Effor Cont, Infant Behaviour Record-Revised Effortful Control; CDI Part l Voca Prod, MacArthur-Bates Communicative Development Inventories Vocab Produced; CDI Part l Voca Und, MacArthur-Bates Communicative Development Inventories Vocab Understand; CDI Part l Total Ges: MacArthur-Bates Communicative Development Inventories Total Gestures.

a Reference value Q3: 11⋅20–12⋅09 (*n* 277).

**Table 5.** Associations between maternal fT3 quintiles and child neurodevelopment outcomes at 20 months of age in the cohort of 1328^a^

	Q1 (*n*262)			Q2 (*n* 269)			Q4 (*n* 269)			Q5 (n269)		
ND test	*β*	95 % Cl	*P* value	*β*	95% Cl	*P* value	*β*	95 % Cl	*P* value	*β*	95 % Cl	*P* value
PDI	−0⋅02	(−2⋅459, 1⋅398)	0⋅590	0⋅008	(−1⋅701, 2⋅095)	0⋅839	0⋅05	(−0⋅605, 3⋅187)	0⋅182	−0⋅01	(−1⋅942, 1⋅878)	0⋅974
MDI	0⋅033	(−1⋅020, 2⋅815)	0⋅359	0⋅045	(−0⋅697, 3⋅070)	0⋅217	0⋅073	(0⋅065, 3⋅843)	0⋅043*	0⋅086	(0⋅397, 4⋅188)	0⋅018*
IBQ Surg	0⋅002	(−0⋅132, 0⋅140)	0⋅954	−0⋅004	(−0⋅141, 0⋅127)	0⋅921	−0⋅01	(−0⋅153, 0⋅116)	0⋅784	−0⋅007	(−0⋅149, 0⋅122)	0⋅847
IBG Neg Affect	0⋅048	(−0⋅062, 0⋅301)	0⋅197	0⋅055	(−0⋅043, 0⋅313)	0⋅138	0⋅098	(0⋅063, 0⋅420)	0⋅008*	0⋅009	(−0⋅158, 0⋅201)	0⋅814
IBQ Effortful Control	0⋅016	(−0⋅102, 0⋅162)	0⋅657	−0⋅054	(−0⋅228, 0⋅032)	0⋅140	−0⋅07	(−0⋅257, 0⋅003)	0⋅056	0⋅023	(−0⋅089, 0⋅174)	0⋅525
CDI Part l Vocab Prod	−0⋅055	(−27⋅807, 3⋅363)	0⋅124	−0⋅05	(−26⋅244, 4⋅340)	0⋅160	−0⋅067	(−30⋅119, 0⋅593)	0⋅060	−0⋅049	(−26⋅419, 4⋅489)	0⋅164
CDI Part l Vocab Understand	−0⋅053	(−28⋅577, 4⋅070)	0⋅141	−0⋅063	(−30⋅192, 1⋅842)	0⋅083	−0⋅075	(−33⋅026, −0⋅858)	0⋅039*	−0⋅043	(−25⋅973, 6⋅400)	0⋅236
CDI Part l Total Gestures	−0⋅032	(−2⋅181, 0⋅832)	0⋅380	−0⋅009	(−1⋅664, 1⋅292)	0⋅805	−0⋅032	(−2⋅159, 0⋅810)	0⋅373	0⋅003	(−1⋅437, 1⋅550)	0⋅941

ND, neurodevelopment test; fT3, free triiodothyronine; Q1: <3⋅32 μmol/l ; Q2: 3⋅32–3⋅58 μmol/l ; Q3: 3⋅59–3⋅84 μmol/l ; Q4: 3⋅85–4⋅40 μmol/l ; Q5: >4⋅41 μmol/l; MDI, Mental Development Index; PDI, Psychomotor Development Index; IBQ Surg, Infant Behaviour Record-Revised Surgency; IBQ Neg Aff, Infant Behaviour Record-Revised Negative Affect; IBQ Effor Cont, Infant Behaviour Record-Revised Effortful Control; CDI Part l Vocab Prod, MacArthur-Bates Communicative Development Inventories Vocab Produced; CDI Part l Vocab Understand, MacArthur-Bates Communicative Development Inventories Vocab Understand; CDI Part l Total Gestures: MacArthur-Bates Communicative Development Inventories Total Gestures.

a Reference value Q3: 3⋅59–3⋅84 (*n* 259).

* Significant value at *P* < 0⋅05 and positive association *β* score.

**Table 6.** Associations between maternal fTSH quintiles and child neurodevelopment outcomes at 20 months of age in the cohort of 1328^a^

	Q1 (*n* 264)			Q2 (*n* 277)			Q4 (*n* 266)			Q5 (*n* 259)		
ND test	*β*	95 % Cl	*P* value	*β*	95 % Cl	*P* value	*β*	95 % Cl	*P* value	*β*	95 % Cl	*P* value
PDI	−0⋅037	(−2⋅867, 0⋅939)	0⋅321	−0⋅016	(−2⋅279, 1⋅474)	0⋅674	−0⋅009	(−2⋅116, 1⋅672)	0⋅818	−0⋅036	(−2⋅986, 0⋅974)	0⋅330
MDI	0⋅018	(−1⋅407, 2⋅396)	0⋅610	0	(−1⋅864, 1⋅883)	0⋅992	−0⋅029	(−2⋅666, 1⋅126)	0⋅426	0⋅028	(−1⋅171, 2⋅691)	0⋅440
IBQ Surg	−0⋅065	(−0⋅258, 0⋅011)	0⋅072	−0⋅041	(−0⋅208, 0⋅056)	0⋅261	−0⋅044	(−0⋅217, 0⋅050)	0⋅222	−0⋅045	(−0⋅222, 0⋅049)	0⋅212
IBG Neg Affect	−0⋅06	(−0⋅329, 0⋅029)	0⋅101	−0⋅018	(−0⋅220, 0⋅132)	0⋅627	−0⋅021	(−0⋅231, 0⋅125)	0⋅561	−0⋅018	(−0⋅226, 0⋅136)	0⋅625
IBQ Effortful Control	0⋅029	(−0⋅078, 0⋅183)	0⋅431	−0⋅018	(−0⋅161, 0⋅096)	0⋅618	0⋅01	(−0⋅111, 0⋅149)	0⋅779	0⋅009	(−0⋅116, 0⋅148)	0⋅814
CDI Part l Vocab Prod	−0⋅001	(−16⋅120, 15⋅487)	0⋅969	0⋅032	(−8⋅629, 22⋅441)	0⋅383	−0⋅028	(−21⋅878, 9⋅515)	0⋅440	0⋅038	(−7⋅447, 24⋅448)	0⋅296
CDI Part l Vocab Understand	0⋅015	(−12⋅910, 19⋅695)	0⋅683	0⋅023	(−10⋅866, 21⋅187)	0⋅528	−0⋅03	(−22⋅916, 9⋅470)	0⋅416	0⋅066	(−1⋅151, 31⋅573)	0⋅068
CDI Part l Total Gestures	0⋅003	(−1⋅433, 1⋅543)	0⋅942	0⋅035	(−0⋅730, 2⋅197)	0⋅326	−0⋅046	(−2⋅457, 0⋅500)	0⋅195	0⋅064	(−0⋅129, 2⋅875)	0⋅073

ND, neurodevelopment test; fTSH, free thyroid-stimulating hormone; Q1: <0⋅82 mIU/l; Q2: 0⋅82–1⋅09 mIU/l ; Q3: 1⋅09–1⋅39 mIU/l; Q4: 1⋅40–1⋅80 mIU/l; Q5: >1⋅80 mIU/l; MDI: Mental Development Index; PDI, Psychomotor Development Index; IBQ Surg, Infant Behaviour Record-Revised Surgency; IBQ Neg Aff, Infant Behaviour Record-Revised Negative Affect; IBQ Effor Cont, Infant Behaviour Record-Revised Effortful Control; CDI Part l Voca Prod, MacArthur-Bates Communicative Development Inventories Vocab Produced; CDI Part l Voca Und, MacArthur-Bates Communicative Development Inventories Vocab Understand; CDI Part l Total Ges: MacArthur-Bates Communicative Development Inventories Total Gestures.

a Reference value Q3: 1⋅09–1⋅39 (*n* 262).

## Discussion

We found no association between maternal thyroid function during pregnancy and children's neurodevelopmental outcomes at 20 months in the Seychelles Child Development Study (SCDS). In this cohort, mothers largely had optimal iodine intakes and thyroid function and only a small number were above or below the reference range. In the Seychelles, mothers appear to regulate thyroid function throughout pregnancy in order to meet the neurodevelopmental needs of their children. However, we did detect some positive associations with neurodevelopmental outcomes. When fT3 was assessed categorically using quintiles, the higher quintile measurements of fT3 (Q4 and Q5) compared to the middle quintile showed improvement in the BSID MDI.

The Seychellois population are proposed to have adequate exposure to dietary iodine through both their high fish consumption, the implementation of iodised salt, and salt used both in the home and within the food industry (i.e. processed items such as bread), with this confirmed by our estimated intake of 233 μg/d^(25)^. Despite this high intake, there was no adverse association with neurodevelopment, an important finding, given the adverse effect of high iodine intakes on thyroid function and thereby offspring neurodevelopment previously reported^(19)^. Nevertheless, in countries with lower fish intake and lack of iodised salt programmes, this may not be the same. As such it remains important for future work to be conducted amongst populations with varying intakes of iodine-rich foods to further investigate the associations between thyroid function and neurodevelopment^(16,17)^.

The importance of adequate maternal thyroid hormone concentrations in relation to optimal cognitive functioning has previously been conducted as part of the SCDS testing. Davidson *et al.* conducted analysis on the NC1 cohort (*n* 300) and found a significant association between maternal TSH concentrations and scores on the Percentage Anticipatory Saccades element of the Fagan Infantest^(24)^. They reported increasing maternal TSH concentrations were associated with a decrease in Percentage Anticipatory Saccades indicating improved performance amongst the assessed children at 5 months of age^(24)^. As the NC2 cohort is larger (*n* 1535) compared to the NC1 cohort (*n* 300), and in tandem with both the high and changing fish consumption amongst the Seychellois cohort, further investigations into maternal thyroid function were justified as part of the present study. Previously conducted studies have reported that there is a delicate balance regarding thyroid hormone measurements and cognitive outcomes, with measurement being time-specific and higher/lower values not necessarily indicative of a simple better or worse relationship^(22,34)^. It is indeed possible that minor imbalances in fT3, as indicated in our quintile analysis (Q3 *v.* Q4 and Q3 *v.* Q5), may still be associated with child neurodevelopment. However, given the lack of association in our primary model, the small number of participants in high and low quintiles and the strength of the association found, further research would be needed to confirm this. Recent work has also highlighted the importance of understanding the natural fluctuation of thyroid hormones throughout pregnancy and the potential implications, positive or negative, that may occur with respect to cognitive outcomes in offspring^(35)^. Several authors have described the role of maternal T4 supplied to the developing foetus as facilitating growth and development of the neuronal cortex, hippocampus, cochlea and cerebellum^(7–9)^. Future studies should give particular attention to the importance of measuring thyroid hormones at earlier stages in gestation such as during the first trimester with respect to neurodevelopment^(35)^.

Our findings are consistent with those of Threapleton *et al.*, who also found no associations between maternal thyroid function and child cognitive outcomes in a large UK cohort. This work suggested that the WHO thresholds may be inadequate and that alternative functional biomarkers for iodine status in pregnancy could be identified and thus may provide additional insight^(36)^. However, the findings of Threapleton *et al.*'s work and the overall conclusion drawn from our research are in contrast with various other studies which have found associations between maternal thyroid status during pregnancy and neurodevelopmental outcomes in offspring. There are reports that offspring born to mothers with low thyroid function in pregnancy had impaired childhood cognitive function with women classified as thyroid deficient during pregnancy having offspring with IQ scores of 4–7 points lower based on the Wechsler Intelligence Scale for Children (WISC)^(37,38)^. On examining the association of maternal TSH and T4 with children's brain volume determined by MRI at age 10 years, both deficient and excessive concentrations throughout pregnancy resulted in smaller brain volume^(39)^. It is worth noting that these studies were observational and conducted in different countries (The Netherlands and USA) which have different dietary patterns, exposure to iodised salt and population ethnicities.

The present study has several strengths, and it is a large sample size in a well-characterised population. The measurements of thyroid hormones were robust, and we were able to account for numerous covariates known to influence neurodevelopment. Moreover, we also measured a range of neurodevelopmental outcomes in the offspring.

Like all epidemiology studies, it also has some limitations. We measured thyroid hormones in the third trimester and were unable to determine the impact that concentrations of the maternal thyroid hormones might have upon neurodevelopment earlier in pregnancy. However, previous research conducted by both Lee *et al.* and Fuse *et. al.* does indicate that thyroidal changes throughout pregnancy are minimal in iodine sufficient areas, such as Japan and Korea as assessed in these studies, and subsequently the Seychelles as analysed in the present study^(40,41)^. Thus, it is plausible that the 28-week time point used as a testing point within our Seychellois cohort, who are known to be iodine sufficient, is potentially not dissimilar to thyroid hormone measurements which could be taken at an earlier time point during gestation, e.g. first trimester. We did not measure the iodine composition of Seychellois fish which could have impacted the accuracy of intake data, nor do we have dietary data for the complete cohort. Additionally, our estimation of iodine intake in the present study was based on food composition data for the UK.

Despite the knowledge that either too high or too low intakes of iodine are associated with poorer childhood neurodevelopmental outcomes in an iodine sufficient population^(19)^, it is important for future investigations to be conducted within cohorts with a different dietary pattern than that of the Seychellois population.

## Conclusion

The overall findings of our longitudinal study show that there are no associations between maternal thyroid function during pregnancy and cognitive outcomes at 20 months in the SCDS. Despite high iodine intakes, which could potentially disrupt maternal thyroid function, there is no evidence of adverse cognitive outcomes in offspring in this high fish-eating population. Secondary analysis revealed positive associations between a higher maternal fT3 concentration and the MDI scores of children when a non-linear relationship with fT3 was tested by quintile analysis. These results point to the delicate balance required of thyroid function during pregnancy for optimal offspring neurodevelopment, results which warrant further advancement by future studies.
